# A machine learning model with human cognitive biases capable of learning from small and biased datasets

**DOI:** 10.1038/s41598-018-25679-z

**Published:** 2018-05-09

**Authors:** Hidetaka Taniguchi, Hiroshi Sato, Tomohiro Shirakawa

**Affiliations:** Department of Computer Science, School of Electrical and Computer Engineering, National Defense Academy of Japan, Yokosuka, 239-8686 Japan

## Abstract

Human learners can generalize a new concept from a small number of samples. In contrast, conventional machine learning methods require large amounts of data to address the same types of problems. Humans have cognitive biases that promote fast learning. Here, we developed a method to reduce the gap between human beings and machines in this type of inference by utilizing cognitive biases. We implemented a human cognitive model into machine learning algorithms and compared their performance with the currently most popular methods, naïve Bayes, support vector machine, neural networks, logistic regression and random forests. We focused on the task of spam classification, which has been studied for a long time in the field of machine learning and often requires a large amount of data to obtain high accuracy. Our models achieved superior performance with small and biased samples in comparison with other representative machine learning methods.

## Introduction

Machine learning has been widely studied and has contributed to technologies used in our everyday life, such as automatic translation, image recognition and spam classification^1^. One notable method of machine learning is supervised learning, which generalizes the concept of a problem from a set of labeled training data. For example, a spam classifier uses training data in the form of a large sample of email texts that have been previously labeled into two classes, spam and ham (i.e., non-spam), to classify new uncategorized emails. The early representative machine learning methods include perceptron^2^, logistic regression (LR)^3^ and the nearest-neighbor rule^4^. Neural networks (NN)^5^ and support vector machine (SVM)^6^ were later proposed based on the perceptron. The other notable machine learning models include naïve Bayes (NB) and random forests (RF)^7^. These machine learning models have been studied for a long time and have shown superior performances across a variety of tasks.

Usually, these models require a large, well-balanced sample dataset to assure prediction accuracy^8^. However, in practice the proportion of real data is often biased. For example, over 90% of emails were identified spam mails in 2012^9^ while the common training datasets such as SpamAssassin^10^ and Ling-Spam^11^ consist only 20–30% of spam labeled data and rests are ham labeled data. Namely, real data is more likely to be imbalanced. Also, spam emails have become difficult to detect since their form dramatically changed^12^. Therefore, the data proportion and property between real data and common datasets as a testbed for machine learning study might have much difference, and we thus consider that there is a strong need for the machine learning model which can deal such situation.

In contrast, humans can generalize a new concept from small and biased samples^13,14^. For example, by seeing a hippopotamus in a zoo for the first time, an infant can obtain a lot of information about the new object: what it looks like, how big it is, and the characteristics that differentiate hippos from other animals. In machine learning, hundreds or thousands of training samples may be required to tackle the same problems^15^. Also, humans do not need a large number of negative samples to learn a positive instance^15^. For example, infants do not need to see elephants, to learn hippos. Namely, human can generalize a new concept from samples of single class, while machine learning requires many data and many labels.

Humans have cognitive biases^16–20^ that effectively support concept acquisition^21,22^. Many researchers have attempted to reproduce human-level concept learning through machine learning^21,23–25^. According to one recent study^22^, two cognitive biases—symmetric bias^22,26^ and mutually exclusive bias^27,28^—can be effectively employed in machine learning tasks. Symmetric bias promotes a tendency of inferring “if *q* then *p*” after convincing that “if *p* then *q*”^29^. For example, if *p* represents “*the weather was rainy*” and *q* represents “*the ground was wet*”, symmetric bias infers “*if the ground was wet* (*q*), *then the weather was rainy a while ago* (*p*)” from “*if the weather was rainy* (*p*), *then the ground was wet* (*q*)”^29^. In practice, such inference can lead to systematic errors^29^ because “*the ground was wet*” can be triggered by other factors such as “*the garden sprinkler sprayed water*”. Therefore, the relationship between “*the weather was rainy*” and “*the ground was wet*” is uncertain in this limited situation. However, this kind of inference can be observed in our everyday life, and this human characteristic is considered to promote faster learning and decision making^22^. Mutually exclusive bias is another tendency in which “if $\overline{p}$ then $\overline{q}$” is inferred after convincing that “if *p* then *q*”, where $\overline{p}$ and $\overline{q}$ are the negations of *p* and *q*. For example, suppose a mother tells her son, “*if you don’t clean up your room*, *then you will not be allowed to play*”. In this sentence, *p* is interpreted as “*not cleaning up a room*” and *q* is interpreted as “*not being allowed to play*”. In this case, her son may also interpret the sentence as “*if I clean up my room*, *then my mom will allow me to play*” (i.e., $\overline{p}\to \overline{q}$), and her son may therefore clean up his room^29^. In this case, the son seems to misunderstand the relationship between the two sentences. However, the communication between the mother and her son seems to be successful, and such conversations are often observed in our daily lives. In linguistic studies, mutually exclusive bias is thought to promote vocabulary growth in children^27–33^. For example, if *p* represents “*being a hippopotamus*” and *q* represents “*being called a hippopotamus*”, the mutually exclusive bias gives a tendency toward inference such that “*if a child recognizes an elephant* ($\overline{p}$), *then the child does not think it is called a hippopotamus* ($\overline{q}$)” is inferred from “*if a child recognizes a hippopotamus* (*p*), *then learns that it is called a hippopotamus* (*q*)”. Thus, mutually exclusive bias is said to prevent the confusion of distinct objects^22^. A number of studies have been conducted on symmetric bias and mutually exclusive bias since they were initially reported. The Δ*P* model^34^ and dual-factor heuristics (DH) model^21^ are examples. The Δ*P* model satisfies the mutually exclusive bias, while the DH model satisfies the symmetric bias^22,29^. As mentioned above, symmetric bias and mutually exclusive bias sometimes lead to incorrect logic but in a rather creative manner. Shinohara *et al*. therefore expected that including both of these biases in a model would yield more human-like inferences^22^. The resulting loosely symmetric (LS) model considers both symmetric and mutually exclusive biases, based on Δ*P* and DH models. In cognitive experiments, the LS model has shown a very high correlation with human inference and can be applied to any tasks that address conditional probability^35^.

In this study, to apply this human-like nature to machine learning tasks, we implemented the LS model within an NB text classifier to learn from small and biased samples. NB has high affinity with LS models since it addresses conditional probabilities. LS model is a well-formulated cognitive model and has a correlation to human inference^35^ and thus we implemented LS model into Naïve Bayes spam classifier to promote concept learning with symmetric bias and mutually exclusive bias.

Although symmetric bias and mutually exclusive bias hold in some conditionals such as “promises” and “threats”^36^, they sometimes lead to incorrect logic. For example, if *p* represents “*the shoe is white*” and *q* represents “*a star is printed on it*”, symmetric bias infers “*if a star is printed on a shoe*, *then the shoe is white*” and mutually exclusive bias infers “*if the shoe is not white*, *then a star is not printed on it*” from “*if the shoe is white*, *then a star is printed on it*”. These inferences are undoubtedly errors. Although the mutually exclusive bias is said to promote word learning in linguistics, it is still under investigation that the symmetric bias and mutually exclusive bias would be the fundamental of cognition, or they work only in limited situations.

However, in practice, symmetric bias and mutually exclusive bias are useful for our task, spam classification. Spam mails often contain spam-likely words such as “Casino” or “Slot”^37^. If *p* represents “*this email contains a spam*-*likely word*”, and *q* represents “*this email is spam*”, symmetric bias gives a tendency toward inference such that “*if this email is spam* (*q*), *then this email contains spam*-*likely word* (*p*)” from “*if this email contains spam*-*likely word* (*p*), *then this email is spam* (*q*)”. Also, mutually exclusive bias gives a tendency toward inference such that “*if this email does not contain spam*-*likely word* ($\overline{p}$), *then this email is ham* ($\overline{q}$)”. In this inference, according to the Equation^38^, we assume *P*(*if p then q*) is equivalent to the conditional subjective probability *P*(*p/q*) in the spam classification task. The number of empirical studies implied the existence of correlation between the conditional “if *p* then *q*” and conditional probability^39,40^. However, Barrouillet and Gauffroy reported that the relationship between *P*(*if p then q*) and *P*(*p/q*) was found only in limited situations^36^. Despite such circumstances, we assume that the Equation is useful for our tasks since the equivalence between the conditionals such as “*if this email is spam* (*q*), *then this email contains spam*-*likely word* (*p*)” and the conditional subjective probability *P* (this email containts spam - likely word|this email is spam) holds. The spam classification is therefore one of the best testbed for our challenge since it involves conditional probability and causal inference between classes and features.

Based on this hypothesis, we developed two models, named loosely symmetric naïve Bayes (LSNB) and enhanced LSNB (eLSNB). These two models have three characteristics that are not involved in traditional NB approaches. Our models (1) permit adjusting feature weights under the condition of feature vectors that belong to each class, while NB classifier assumes that every feature in a text is conditionally independent, (2) address a set of *n*-dimensional feature vectors to calculate a posterior probability, while NB addresses only one feature vector from one class and (3) retain the simplicity and processing speed of NB classifiers while offering superior performance. These characteristics effectively adjust feature weights within a restricted data distribution.

The contribution of this paper is directed to the both fields of machine learning and cognitive science. We investigated the efficiency of cognitive model on machine learning tasks: how cognitive biases support machine learning with small and biased data. Our contribution is not limited to the spam classification task. Rather, we studied how human cognitive biases and machine learning models can be integrated to realize fast learning from small and biased data, namely, imitating human-level concept learning. Our models yielded superior performances compared with conventional machine learning models when learning was performed based on small and biased samples.

## Methods

### Naïve Bayes

NB is a generative model based on Bayes’ theorem^41^ and is often used for text classification^42^. In spam classification, each message is characterized as an *n*-dimensional word feature vector $W=\langle {w}_{1},{w}_{2},\mathrm{...},{w}_{n}\rangle$ that is predefined to belong to class $C=\langle spam,ham\rangle ,{c}_{i}\in C$, where *ham* is non-spam. The posterior probability that $W$ belongs to ${c}_{i}$ is given as defined in equation (1).

1 $$P({c}_{i}|W)=\frac{P({c}_{i})P(W|{c}_{i})}{P(W)}=\frac{P({c}_{i})\prod _{j=1}^{|W|}P({w}_{j}|{c}_{i})}{P(W)}$$

*P*(*W*) is called “evidence” and takes the same value for all classes and does not affect the relative values of their probability^43^. It can be ignored, as in equation (2).

2 $$P({c}_{i}|W)\propto P({c}_{i})\prod _{j=1}^{|W|}P({w}_{j}|{c}_{i})$$

The NB classifier assumes that every feature in a text is conditionally independent and that each distribution is estimated as a one-dimensional distribution^1^. In practice, this assumption is unrealistic because words such as “money” and “casino” are likely to co-occur in the same text. However, this assumption of conditional independence greatly simplifies the calculation and yields superior performance in text classification.

### Loosely symmetric naïve Bayes

We implemented the LSNB model based on the naïve Bayes classifier. Table 1 shows the 2 × 2 contingency table of the LS model, where *a*, *b*, *c* and *d* are the frequencies or joint probabilities of *p* and *q*.

**Table 1:** 2 × 2 Contingency table of the LS model.

	*q*	$\overline{q}$
*p*	*a*	*b*
$\overline{p}$	*c*	*d*

The LS model estimates the strength of the relation between *p* and *q* as defined in equations (3–6).

3 $$LS(q|p)=\frac{a+\frac{bd}{b+d}}{a+b+\frac{ac}{a+c}+\frac{bd}{b+d}}$$

4 $$LS(\bar{q}|p)=\frac{b+\frac{ac}{a+c}}{a+b+\frac{ac}{a+c}+\frac{bd}{b+d}}$$

5 $$LS(p|q)=\frac{a+\frac{cd}{c+d}}{a+c+\frac{ab}{a+b}+\frac{cd}{c+d}}$$

6 $$LS(\bar{q}|\bar{p})=\frac{d+\frac{ac}{a+c}}{c+d+\frac{ac}{a+c}+\frac{bd}{b+d}}$$

**Figure 1:**
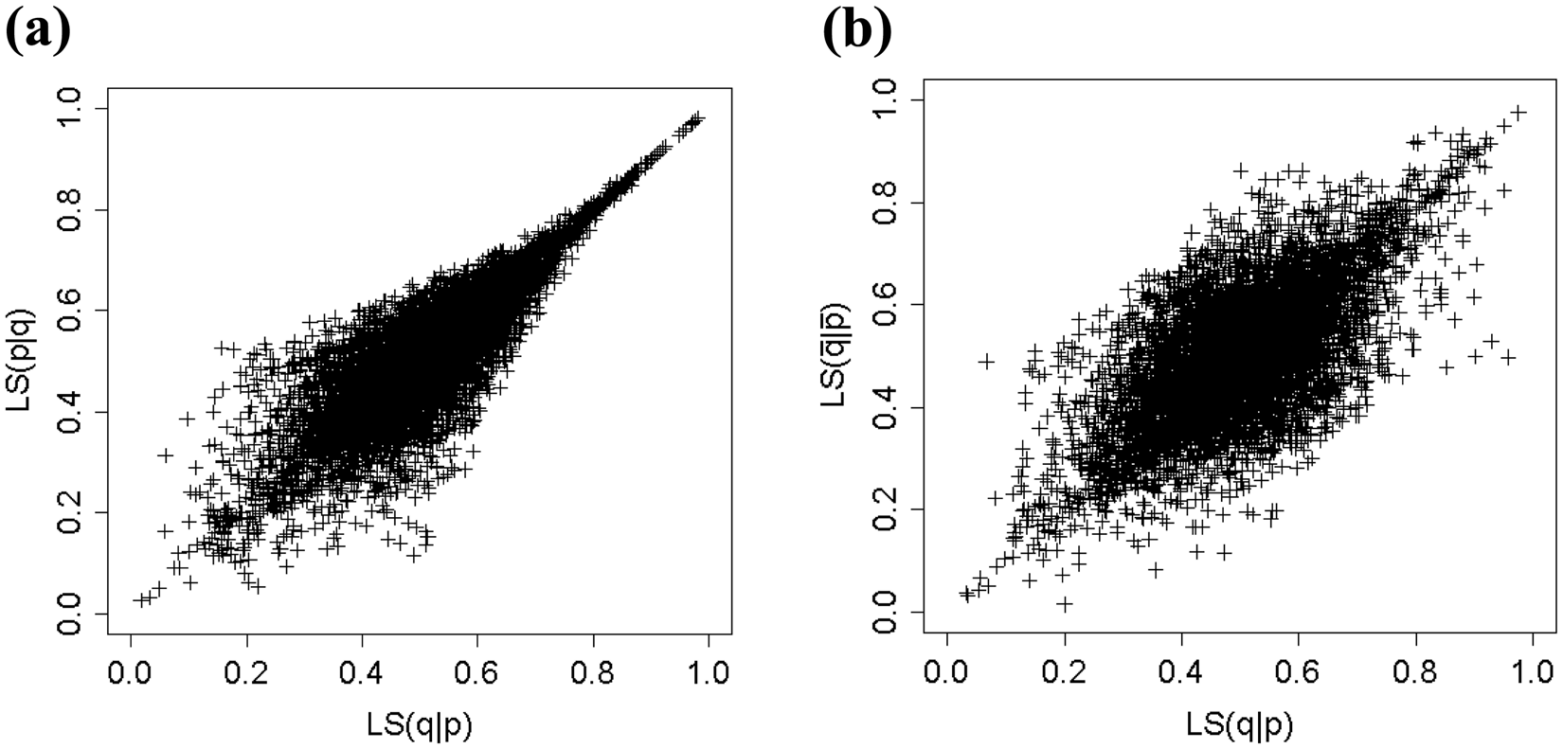
(**a**) Relation between $LS(q|p)$ and $LS(p|q)$. (**b**) Relation between $LS(q|p)$ and $LS(\bar{q}|\bar{p})$.

Though the equation may appear somewhat complicated, it is just a modified conditional probability. For example, equation (3) is a conditional probability modified by including the terms $\frac{ac}{a+c}$ and $\frac{bd}{b+d}$. If both terms equal zero, the model is equivalent to conditional probability, namely, there is no bias. If *b* = *c* is satisfied, equations (3) and (5) are always equivalent and the model has a complete symmetric bias. Additionally, if *a* = *d* and *b* = *c* are simultaneously satisfied, equations (3 and 5) and (6) are equivalent and the model has complete symmetric and mutually exclusive biases. Figure 1, which shows the relation between *LS*(*q*|*p*) and *LS*(*p*|*q*) as well as the relation between *LS*(*q*|*p*) and $LS(\bar{q}|\bar{p})$, demonstrates this. The data points in the figure are randomly generated by uniformly estimating *a*, *b*, *c* and *d* from [0, 1]. If the bias is complete, then *LS*(*q*|*p*) = *LS*(*p*|*q*) and $LS(q|p)=LS(\bar{q}|\bar{p})$ hold, and the graphs have a positive and proportional relationship. If there is no bias, there is no correlation between *LS*(*q*|*p*) and *LS*(*p*|*q*) or between *LS*(*q*|*p*) and $LS(\bar{q}|\bar{p})$, so the distribution of the plots in Fig. 1 would be random. The distributions of the plots in Fig. 1 show an intermediate shape: a hybrid proportional and random distribution. It seems trivial, however if the model always completes symmetric bias or mutually exclusive bias, the model would be illogical and does not show similarity to human inference. Δ*P* and DH models always complete either bias and conditional probability does not involve any biases^22,29^. Meanwhile, the LS model flexibly adjusts the weights of each type of bias using the terms $\frac{ac}{a+c}$ and $\frac{bd}{b+d}$. Namely, the LS model exists in an intermediate state between complete bias and no bias. Although the LS model exhibits the intermediate states of symmetry and mutually exclusivity as shown in Fig. 1, its theoretical mechanism is still under investigation. However, this model showed higher correlation to human inference compared to other cognitive models such as Δ*P* and DH models^29^.

In order to apply the formula to an NB approach, the 2 × 2 contingency table is arranged as in Table 2 and LSNB is calculated as in equations (7–10).

7 $$a=P({w}_{j}|{c}_{i})$$

8 $$b=P(\overline{{w}_{j}}|{c}_{i})$$

9 $$c=P({w}_{j}|\overline{{c}_{i}})$$

10 $$d=P(\overline{{w}_{j}}|\overline{{c}_{i}})$$

**Table 2:** Contingency table used in the LSNB model.

	*w*_*j*_	$\overline{{w}_{j}}$
*c*_*i*_	*a*	*b*
$\overline{{c}_{i}}$	*c*	*d*

Above, $a=P({w}_{j}|{c}_{i})$ is the probability that word *w*_*j*_ co-occurs in class *c*_*i*_, and $b=P(\overline{{w}_{j}}|{c}_{i})$ is the probability that word *w*_*j*_ does not co-occur in class *c*_*i*_. Similarly, $c=P({w}_{j}|\overline{{c}_{i}})$ and $d=P(\overline{{w}_{j}}|\overline{{c}_{i}})$ are the probabilities of co-occurrence in the presence or absence, respectively, of *w*_*j*_ in class $\overline{{c}_{i}}$. Each probability is given by equations (7–10), and the modified weight of word *w*_*j*_ in class *c*_*i*_ is calculated as in equations (11–13).

11 $${P}_{LS}({w}_{j}|{c}_{i})=\frac{a+\frac{bd}{b+d}}{a+b+\frac{ac}{a+c}+\frac{bd}{b+d}}$$

12 $${P}_{LS}(W|{c}_{i})=\prod _{j=1}^{|W|}{P}_{LS}({w}_{j}|{c}_{i})$$

13 $${P}_{LS}({c}_{i}|W)=P({c}_{i}){P}_{LS}(W|{c}_{i})$$

For example, if some words such as “money” and “casino” were only observed in *spam* labeled texts much more frequently from *ham* labeled texts, these words should be considered *spam* related words, and vice-versa; the settings above reflect such situation. The main difference between NB and LSNB is the way in which they calculate the posterior probability; in the NB calculation process, the likelihood $P(W|{c}_{i})$ is given by $P({w}_{j}|{c}_{i})=a/(a+b)$, while the LSNB method produces the likelihood ${P}_{LS}({w}_{j}|{c}_{i})$ using *a*, *b*, *c* and *d*.

### Enhanced loosely symmetric naïve Bayes

We further modified the LSNB model to develop the eLSNB model, which includes word density, defined in equation (14).

14 $$WD({c}_{i},{w}_{j})=\frac{N({c}_{i},{w}_{j})}{\sum _{k=1}^{|W|}N({c}_{i},{w}_{k})}$$

In the above formula, $N({c}_{i},{w}_{j})$ is the number of times word *w*_*j*_ occurs in class *c*_*i*_ and $WD({c}_{i},{w}_{j})$ is the word density. The word density is used as a confidence measure in many text classification applications^44^. We developed this model to more optimally adjust the weights of each feature. For example, if word *w*_*j*_ is frequently observed in *spam* texts but infrequently in *ham* texts, then word *w*_*j*_ should be more strongly considered to be a *spam*-related word. The reason why we employed word density information into eLSNB is that external bias can be effectively employed in some practical cases. The causal relationship is sometimes difficult to estimate from observed raw data. In such a condition, the eLSNB model introduces bias into the features and flexibly modifies its weight as shown in equations (15–18).

15 $$a=P({w}_{j}|{c}_{i})WD({c}_{i},{w}_{j})$$

16 $$b=P(\overline{{w}_{j}}|{c}_{i})WD(\overline{{c}_{i}},{w}_{j})$$

17 $$c=P({w}_{j}|\overline{{c}_{i}})WD(\overline{{c}_{i}},{w}_{j})$$

18 $$d=P(\overline{{w}_{j}}|\overline{{c}_{i}})WD({c}_{i},{w}_{j})$$

After the weight modifications, the eLSNB model calculates the likelihood and the posterior probability as in equations (11–13).

### Email corpus

We used two publicly available English email corpora in the experiment. The SpamAssassin^10^ corpus consists of 3900 *ham* messages and 1897 *spam* messages, and 33% of the sample data were *spam*. The Ling-Spam^11^ corpus consists of 2412 *ham* messages and 481 *spam* messages. We used the *lemm* version (texts are lemmatized) of the Ling-Spam corpus for the experiment, and 17% of the messages were *spam*.

### Experimental Settings

We conducted two experiments with different percentages of *spam* and *ham* messages in the learning phase, using seven classification models: SVM, NN, LR, RF, NB, LSNB and eLSNB. The SVM classifier was used with a Gaussian kernel, which is common for text classification with $cost=0.1$, $gamma=0.1$. We used a three-layered NN with a sigmoid function, which is common for binary classification. The number of nodes in a hidden layer was 10. The number of nodes seems few, but we found this value is suitable for the following experiments. The LR used binominal regression with *α* = 0.1. RF used 300 trees, and the number of features for the decision split was the square root of the dimensions of the feature space. For the experimental settings of the NB, LSNB and eLSNB models, we set prior probabilities to be equal for each class, namely, $P(spam)=0.5$ and $P(ham)=0.5$, to avoid any initial asymmetry. Half of the whole dataset was used as test data. The parameters of each model were decided after some trials and chosen best values for the experiments.

In the following experiments, we used only biased and skewed numbers of training data. In Exp. 1-1, the same number of *spam* and *ham* messages were used as training data, where $spam=t\ast 40$ and $ham=t\ast 40$, for $[t|1\le t\le 6]$, with 50% *spam*. In Exp. 1-2, there were differing numbers of *spam* and *ham* messages used as training data, with $spam=t\ast 24$ and $ham=t\ast 40$, for $[t|1\le t\le 10]$, producing 38% *spam*. In Exp. 1-3, 17% of messages were *spam* with $spam=t\ast 8$ and $ham=t\ast 40$, for $[t|1\le t\le 30]$. In Exp. 2, the amount of training data belonging to either class was set to a constant value. In Exp. 2-1, the size of the *spam* training data was 25 or 100 messages as a constant parameter while the number of *ham* training messages was $ham=t\ast 40$, for $[t|1\le t\le 6]$. Therefore, only the number of *ham* sample messages increased, while the number of *spam* sample messages was constant. In Exp. 2-2, the number of *ham* training data messages was 25 or 100 as a constant parameter and the number of *spam* training data messages was $spam=t\ast 40$, for $[t|1\le t\le 6]$. The settings used for Exp. 2-2 were the inverse of those used in Exp. 2-1. In particular, Exp. 1 was an investigation how biased data would affect machine learning model and Exp. 2 was an investigation how small data also affects the performances.

Before the experiments were conducted, we eliminated punctuation, numerals and stop words^45^ from email texts as well as any word features that were observed only once. Stop words are English words commonly observed in any general text, such as “this” and “you”. These words do not affect classification and are often eliminated before the training phase. Furthermore, according to the theory of burstiness^46,47^, words related to the text content tend to be observed more than once. Thus, we eliminated words from the feature vector that were observed less than twice.

All experiments were implemented using R (https://www.r-project.org). We used the e1071 package for SVM, the nnet package for NN, the glmnet package for LR, and the randomForest package for RF. NB, LSNB and eLSNB models were implemented within the R statistical computing environment using custom scripts.

## Results

We compared the performance of NB, LSNB, eLSNB, SVM, NN, LR and RF on spam classification. The purpose of the task was to classify texts into one of two classes, *spam* and *ham*. We used two mail corpora, SpamAssassin and Ling-Spam, in the following experiments.

### Experiment 1

In the following experiments, we varied the percentage of *spam* training data in each experiment and compared the *spam* classification accuracy, *ham* classification accuracy and F-measure.

### Experiment 1-1

**Figure 2:**
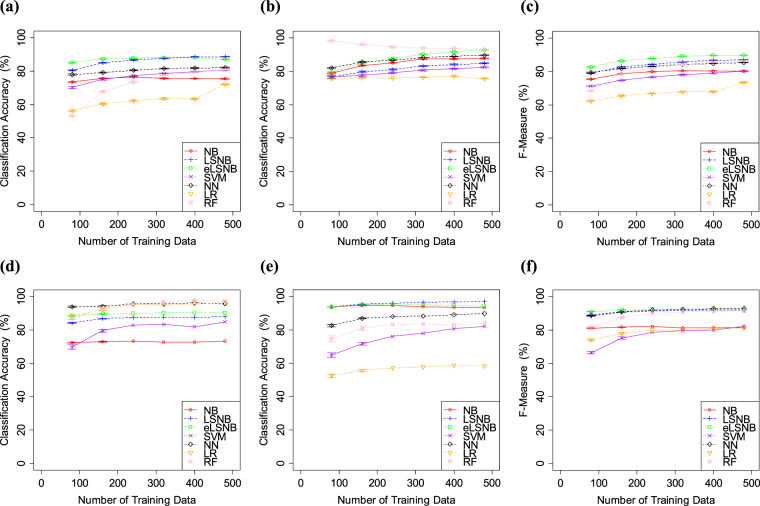
(**a**) *spam* classification accuracy, (**b**) *ham* classification accuracy and (**c**) F-measure values for SpamAssassin. (**d**) *spam* classification accuracy, (**e**) *ham* classification accuracy and (**f**) F-measure values for Ling-Spam. Error bars indicate the standard error.

The results of Exp. 1-1 are shown in Fig. 2. Overall, eLSNB, LSNB, NN and RF methods achieved higher classification accuracy for *spam* classification. NB did not improve the *spam* classification accuracy through the experiment and showed relatively lower accuracy. In the *ham* classification, eLSNB, LSNB, NB and NN showed higher classification accuracy compared to the other models. LR did not improve the *ham* classification accuracy through the experiment and was the worst among all the models.

In this experiment, the total number of training data was less than 500. Therefore, each classification model was expected to have difficulty in optimizing the proper weights for each feature. However, the eLSNB, LSNB, NN and RF yielded higher F-measure scores. Additionally, eLSNB and LSNB models exhibited improved *spam* and *ham* accuracies compare to the NB base model. Overall, eLSNB performed the best in terms of F-measure, supported by its use of the LS model and word density information for optimizing the feature weights from a small number of samples.

### Experiment 1-2

**Figure 3:**
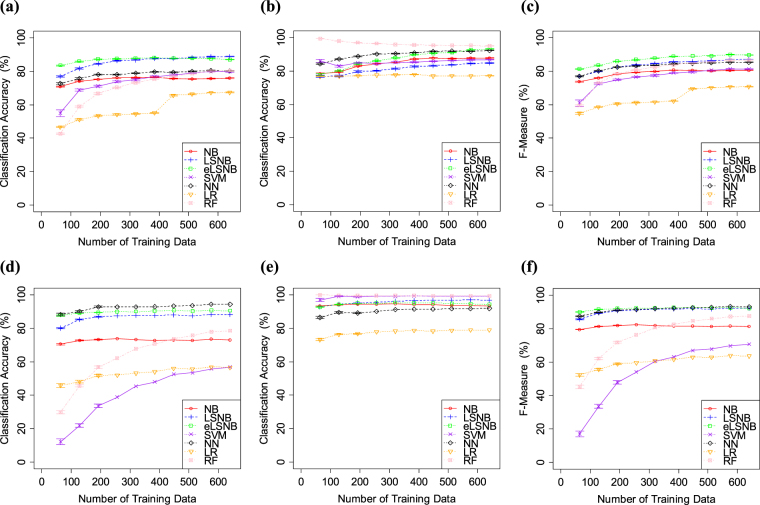
(**a**) *spam* classification accuracy, (**b**) *ham* classification accuracy and (**c**) f-measure values for spamassassin. (**d**) *spam* classification accuracy (**e**) *ham* classification accuracy and (**f**) f-measure values for ling-spam. Error bars indicate the standard error.

The results of Exp. 1-2 are shown in Fig. 3. Although the percentage of *spam* training data in this experiment was higher than that in Exp. 1-1, almost all the models yielded similar results. For example, NB, LSNB, eLSNB and NN models performed similarly throughout Exp. 1. Meanwhile, RF, LR and SVM showed some trade-offs between *spam* and *ham* accuracies. These models improved *ham* classification accuracy and decreased *spam* classification accuracy compared with the results in Exp. 1-1. Therefore, the performances of RF, LR and SVM were affected by the *spam* percentage and expected to have some sensitivity to imbalanced data.

### Experiment 1-3

**Figure 4:**
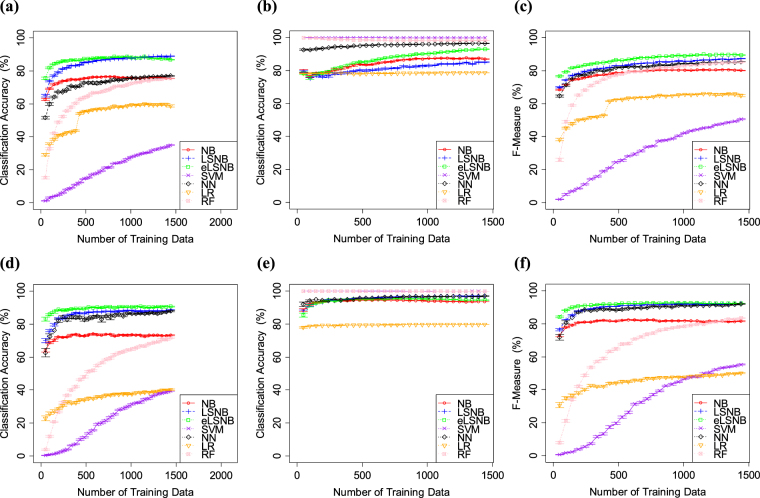
(**a**) *spam* classification accuracy, (**b**) *ham* classification accuracy and (**c**) F-measure values for SpamAssassin. (**d**) *spam* classification accuracy, (**e**) *ham* classification accuracy and (**f**) F-measure values for Ling-Spam. The error bars indicate the standard errors.

The results of Exp. 1-3 are shown in Fig. 4. SVM and RF showed nearly perfect accuracy in *ham* classification, followed by NN and eLSNB, in descending order. LR and NB showed relatively lower *ham* classification accuracy. Additionally, SVM, RF and NN showed improved *ham* classification accuracy relative to Exps 1-1 and 1-2. However, *spam* classification accuracies of SVM, RF and LR were relatively lower than those of other models. SVM, RF and NN also exhibited decreased *spam* classification accuracy in Exps 1-1 and 1-2. Additionally, the trade-offs of RF, LR and SVM became wider in Exp. 1-3 relative to Exp. 1-2. Furthermore, NN showed a trade-off that was not observed in Exp. 1-1 or 1-2. Meanwhile, the eLSNB, LSNB and NB did not show such a trade-off and produced higher *spam* classification accuracy. NB, LSNB and eLSNB did not appear to be affected by changes in the class distributions. The proposed models each outperformed the NB base model. Thus, eLSNB and LSNB approaches had some advantage under the biased class distribution in comparison with other models, somewhat resembling the fast learning that is characteristic of humans. Overall, Exp. 1 showed that the LSNB and eLSNB methods simply produced higher classification accuracy than NB and had the highest performance in terms of F-measure.

### Experiment 2

In the following experiments, the number of training data of either class was predefined to be a constant value.

### Experiment 2-1

**Figure 5:**
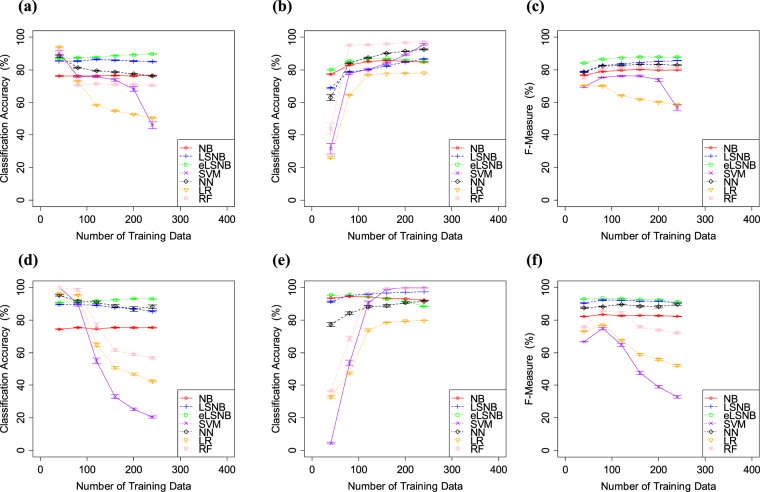
(**a**) *spam* classification accuracy, (**b**) *ham* classification accuracy and (**c**) F-measure values for SpamAssassin. (**d**) *spam* classification accuracy, (**e**) *ham* classification accuracy and (**f**) F-measure values for Ling-Spam. The number of *spam* training data points was predefined at 100. Error bars indicate the standard error.

**Figure 6:**
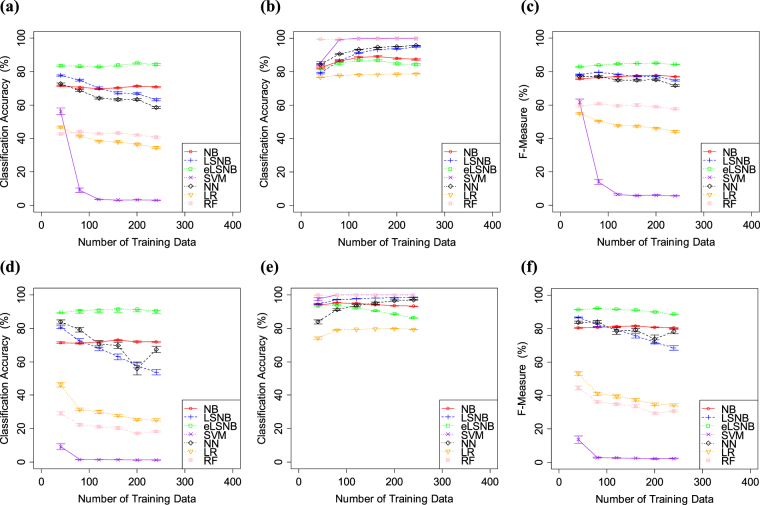
(**a**) *spam* classification accuracy, (**b**) *ham* classification accuracy and (**c**) F-measure values for SpamAssassin. (**d**) *spam* classification accuracy, (**e**) *ham* classification accuracy and (**f**) F-measure values for Ling-Spam. The number of *spam* training data points was predefined to 25. Error bars indicate the standard error.

The results of Exp. 2-1 are shown in Fig. 5 (the number of *spam* training data was predefined as 100) and Fig. 6 (the number of *spam* training data was predefined as 25). When the *spam* training data contained 100 *spam* messages, all models showed increased *ham* classification accuracy. Meanwhile, NN, LR, RF and SVM showed decreased *spam* classification accuracy as the training data increased in number. Thus, these models showed some sensitivity to the data distribution, owing to the lack of *spam* relative to *ham* in the training data. If the number of *spam* messages in the training data is large enough relative to *ham*, each machine learning models is able to estimate the proper weights for each feature. However, in this experiment, the feature distributions between *spam* and *ham* were strongly biased. NN, LR, RF and SVM could not properly weight each feature and *spam* classification accuracy decreased as the number of *ham* training data increased. Meanwhile, LSNB and eLSNB did not decrease either *spam* or *ham* classification accuracies. Overall, eLSNB produced superior results in terms of F-measure.

When the number of *spam* training data was predefined as 25, RF, SVM and NN performed better in *ham* classification. However, as in Exp. 2-1, these models did not show superior results on *spam* classification. In particular, SVM showed higher *ham* classification accuracy, though the *spam* classification accuracy was the worst among all the models. The NN, RF and LR also showed similar trade-offs. LSNB was also affected by the biased sample data, which was not observed in Exp. 1. Although LSNB produced higher *ham* scores, the *spam* classification accuracy decreased as the sample dataset increased in size. Meanwhile, NB and eLSNB did not show such trade-offs. Therefore, LSNB seems to fail to adjust the effectiveness of its symmetry and mutually exclusive biases caused by the strongly imbalanced data distribution, even though it produced superior results in Exp. 1. Meanwhile, eLSNB did not show such a trade-off and produced relatively better *spam* and *ham* classification accuracies. Overall, eLSNB showed the best classification results in terms of F-measure.

### Experiment 2-2

**Figure 7:**
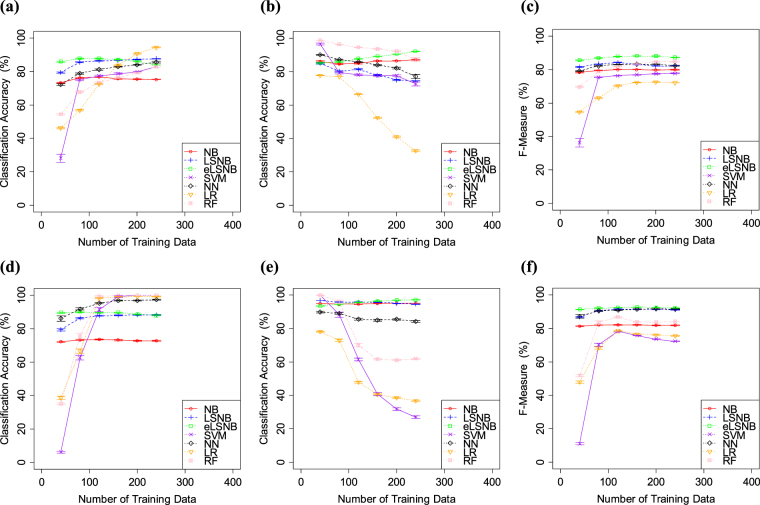
(**a**) *spam* classification accuracy, (**b**) *ham* classification accuracy (**c**) F-measure values for SpamAssassin. (**d**) *spam* classification accuracy, (**e**) *ham* classification accuracy and (**f**) F-measure values for Ling-Spam. The number of *ham* training data points was predefined to 100. Error bars indicate the standard error.

**Figure 8:**
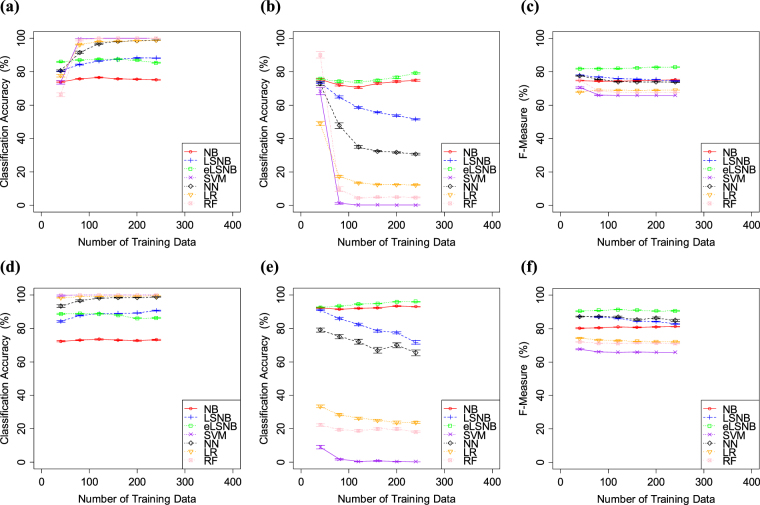
(**a**) *spam* classification accuracy, (**b**) *ham* classification accuracy and (**c**) F-measure values for SpamAssassin. (**d**) *spam* classification accuracy, (**e**) *ham* classification accuracy and (**f**) F-measure values for Ling-Spam. The number of *ham* training data points was predefined as 25. Error bars indicate the standard error.

The results of Exp. 2-2 are shown in Fig. 7 (where the number of *ham* training data was predefined to 100) and Fig. 8 (where the number of *ham* training data was predefined to 25). When there were 100 *ham* training data, almost all the models increased *spam* classification accuracy as the size of the training data increased. Meanwhile NN, SVM, LR and RF models decreased in *ham* classification accuracy through the experiment. This suggests that these models also showed some sensitivity, as seen in the results of Exp. 2-1.

When there were 25 *ham* training data points, eLSNB and NB had superior performance in *ham* classification. Meanwhile, NN, LR, RF and SVM did not show higher performance, in spite of these models having had higher *ham* classification accuracy in Exps 1 and 2-1. Also, NN, LR, SVM and RF had the best performance in *spam* classification. The data proportions between Exps 2-1 and 2-2 were symmetric, and therefore the *spam* classification results of Exp. 2-1 and *ham* classification results in Exp. 2-2 were similar. The results might not show such symmetry if the data properties or feature distributions were very different between the *spam* and *ham* training data. Since most models showed trade-offs as the dataset increased in size, these models had some sensitivities to imbalances in the data distributions. As Exps 1 and 2 showed, SVM, LR, RF and NN were strongly affected by the data ratio. NB did not show such strong trade-off, but its performance was relatively lower. The proposed LSNB model showed a trade-off in Exp. 2, and the bias adjustment of the model failed somewhat in some cases. Meanwhile, eLSNB overcame this weakness and word density information helped to prevent the problematic data sensitivity. Overall, eLSNB had the highest F-measure values.

## Discussion

The present study tested the performance of NB, SVM, NN, LR and RF machine learning methods against our models, designated LSNB and eLSNB, using small and biased samples. We focused on the classic spam classification task, which has been studied for a long time in the field of machine learning. The data proportion and contexts between real spam mail data and common spam classification datasets have much difference, and the machine learning model which can deal such situation is strongly needed. The conventional algorithms, such as NB, NN, SVM, LR and RF, often require a large amount of well-balanced sample data to assure prediction accuracy in tasks such as spam classification. In contrast, humans can generalize a new concept from a small number of samples, even if the composition of the samples is imbalanced^13–15^. Some researchers claim that human beings have cognitive biases^16–20^ and that these biases facilitate concept learning from small and biased samples^21,22^. We developed LSNB and eLSNB based on this hypothesis and attempted to reproduce this small and biased sample scenario properly as a machine learning task. The difference between NB and our models is that LSNB and eLSNB include two additional terms $\frac{ac}{a+c}$ and $\frac{bd}{b+d}$, which modify the probabilities of the models. As shown in the Methods section, these two terms adjust the effectiveness of symmetric bias and mutually exclusive bias; in other words, they promote concept learning, but do not always make correct inferences.

In the experiments, we tested the models using different percentages of *spam* and *ham* data in the learning phase to investigate how model behaviors changed according to changes in the feature distribution. In Exp. 1-1, we used the same numbers of *spam* and *ham* training data points, and Exps 1-2 and 1-3 used less *spam* and more *ham* data (33% *spam* in Exp. 1-2 and 17% in Exp. 1-3). These three experiments were investigations of how biased data would affect the performances of machine learning models.

In Exp. 1-1, every model showed higher classification accuracy on *spam* and *ham* and most models increased their performance with the incensement of data size. However, SVM, LR and NB showed relatively lower *spam* and *ham* classification accuracies. The total number of training data in this experiment was less than 500. Therefore these models did not perform well from such a small number of training data. In contrast, LSNB and eLSNB simply improved upon the performance of the NB base model, producing superior results. The class distribution of this experiment was equal between *spam* and *ham*. Therefore trade-off was not observed from every model.

In Exp. 1-2, SVM, LR and RF showed trade-offs between *spam* and *ham* classifications. The *spam* classification accuracies of these models were relatively lower at the initial stage and gradually increased throughout the experiment. Meanwhile their *ham* classification performances merely increased and showed similar F-measure scores as shown in Exp. 1-1. Although RF showed higher F-measure scores, its sensitivity to class distributions was observed. In practice, NB, SVM, LR and RF often require a large amount of training data to assure the prediction accuracy. However, we only used limited number of training data in this study. These models thus showed less ability of learning from limited number of data. Meanwhile eLSNB, LSNB and NN did not show trade-offs and kept higher classification performances.

In Exp. 1-3, SVM and RF showed bigger trade-offs. These models showed almost perfect *ham* classification performances, even when the number of training data was small. However, their *spam* accuracies were very low. Although their *spam* accuracies increased throughout the experiment, these models did not perform as well as other models. Furthermore, NN did not show trade-offs in Exps 1-1 and 1-2, however, its *spam* accuracy decreased and *ham* classification performances were merely increased. This fact suggests NN also suffered from small biased training data. eLSNB, LSNB and NB did not show such trade-offs and showed higher *spam* and *ham* classification performances. The *spam* accuracy of NB was relatively lower. Meanwhile LSNB and eLSNB increased their performance compare to its base model NB.

In Exp. 1, each model showed interesting tendency for the data ratios. NN, SVM, LR and RF showed trade-offs between *spam* and *ham* classification accuracies. In particular, the trade-offs of these models became bigger and bigger as the *spam* percentage decreased. Therefore these models exhibited some sensitivity to the feature distribution and their accuracies have widely fluctuated. NB did not show such a trade-off; however, its classification performance was relatively low. In contrast, LSNB and eLSNB simply improved upon the performance of the NB base model, producing superior results. LSNB and eLSNB adjust feature weights using feature vectors for each class, while NB simply calculates the product of the conditional probability. We consider that this modification yielded better learning from small and biased samples, and eLSNB produced the best performance in terms of its F-measure. The eLSNB model is a modified version of the LSNB model that uses word density information. This modification successfully improved the learning process.

In Exp. 2, we investigated the effect of more imbalanced sample distributions on machine learning models. We predefined the number of training data of either class at a constant value, i.e., 100 or 25. Therefore, the disparity in the number of training data between *spam* class and *ham* class messages became progressively wider throughout the experiment. Accordingly, the feature distribution of the training data was strongly imbalanced. In this experiment we focused on how small data affect the performances of machine learning models.

In Exp. 2-1, all models had strong trade-offs throughout the experiments and decreased in accuracy as the size of the training data set increased, except for the NB and eLSNB models. For example, the *spam* classification performances of SVM, LR, RF and NN decreased significantly as the size of *ham* training data increased. At the initial stage of the experiment, these models had lower *ham* performances and higher *spam* performances. If the models were able to optimize their performance under an imbalanced data distribution, such a decrease in accuracy would not be observed. As the data proportions of Exps 2-1 and 2-2 were symmetric, the *spam* classification results in Exp. 2-1 and *ham* classification results in Exp. 2-2 were similar. This fact suggests that the composition of feature distributions were symmetric between Exps 2-1 and 2-2. For example, if the *spam* data is easier to classify than *ham* data, the results would be asymmetrical and vice-versa. Therefore, there is no initial asymmetry between *spam* and *ham* training data. We consider that these trade-offs were not caused by the contents of the corpus, but rather the difference in the number of training data points belonging to each class—in other words, the imbalanced data distribution. SVM, LR, RF and NN were strongly affected by this factor. Also, our LSNB model showed a trade-off even though its NB base model did not decrease in performance through the experiment. We cannot explain the exact reason why LSNB showed such a trade-off, but we assume that LSNB may not fully adjust to the effects of symmetric and mutually exclusive biases. Although NB did not exhibit strong trade-off, its performance was relatively low. Additionally, we roughly estimate that there is a difference in characteristics between the NB and NN; NB did not show a trade-off but its classification performance was relatively low, while NN showed higher performance in terms of F-measure, but it had a strong trade-off. In contrast, eLSNB did not show such a trade-off and consistently produced the best F-measure. The inclusion of word density information in the eLSNB model appeared to overcome the data sensitivity of the base LSNB model. In practice, as a form of eLSNB, word density strengthened the contraposition of feature values in the 2 × 2 contingency table. As previous studies have indicated, human cognitive biases play a key role in the ability to learn from small and biased samples. However, we assume that human cognitive biases themselves are not powerful enough to produce human-level concept leaning, and additional biases, such as word density, may be needed. Since the relationship between cause and effect is sometimes difficult to infer from observed raw data, external biases may promote concept learning in models, even if it is not derived from human cognition directly.

In conclusion, we developed LSNB and eLSNB models that include symmetric bias and mutually exclusive bias by implementing the LS model into a base NB model. These novel models were successful, yielding higher performance compared with existing representative machine learning algorithms with small and biased samples. Our models seem to have reproduced the ability of human learning to some extent. In future research, we will investigate the relationship between conditional probability, human cognitive bias, the effectiveness of external bias and how these factors interact in the learning process in order to realize human-level concept learning.

## References

[1] Alpaydin, E. Introduction to machine learning (MIT press, 2014).

[2] Rosenblatt F (1958). The perceptron: a probabilistic model for information storage and organization in the brain. Psychological review.

[3] Cox D (1958). The regression analysis of binary sequences (with discussion). Journal of the royal statistical society: series b.

[4] Cover T, Hart P (1967). Nearest neighbor pattern classification. IEEE transactions on information theory.

[5] Werbos, P. J. Beyond regression: new tools for prediction and analysis in the behavioral sciences. Doctoral thesis, Harvard University (1975).

[6] Vapnik, V. The nature of statistical learning theory (Springer, 1963).

[7] Breiman L (2001). Random forests. Machine learning.

[8] T. M. Mitchell Machine learning (McGraw Hill, 1997).

[9] Rao JM, Reiley DH (2012). The economics of spam. Journal of Economic Perspectives.

[10] Mason, J. SpamAssassin Public Corpus, http://spamassassin.apache.org/publiccorpus (2003).

[11] Androutsopoulos, I., Koutsias, J., Chandrinos, K. V., Paliouras, G. & Spyropoulos, C. D. An evaluation of naive Bayesian anti-spam filtering. arXiv preprint cs/0006013 (2000).

[12] Goodman J, Cormack GV, Heckerman D (2007). Spam and the ongoing battle for the inbox. Communications of the ACM.

[13] Lake BM, Salakhutdinov R, Tenenbaum J (2015). Human-level concept learning through probabilistic program induction. Science.

[14] Lake, B. M., Ullman, T. D., Tenenbaum, J. B. & Gershman, S. J. Building machines that learn and think like people. *Behavioral and Brain Sciences* **40** (2017). 10.1017/S0140525X16001837 27881212

[15] Tenenbaum, J. Bayesian modeling of human concept learning. *Advances in neural information processing system* 59–65 (1999).

[16] Kahneman, D. Thinking, fast and slow (Macmillan, 2002).

[17] Tversky E, Kahneman D (1973). Availability: a heuristics for judging frequency and probability. Cognitive psychology.

[18] Tversky E, Kahneman D (1974). Judgement under uncertainty: heuristics and biases. Science.

[19] Feldman J (2000). Minimization of boolean complexity in human concept learning. Nature.

[20] Goodman ND, Tenenbaum JB, Feldman J, Griffiths TL (2008). A rational analysis of rule‐based concept learning. Cognitive science.

[21] Hattori M, Oaksford M (2007). Adaptive non‐interventional heuristics for covariation detection in causal induction: model comparison and rational analysis. Cognitive science.

[22] Shinohara S, Taguchi R, Katsurada K, Nitta T (2007). A model of belief formation based on causality and application to n-armed bandit problem. T. Jpn. Soc. A. I..

[23] Lake B, Salakhutdinov R, Gross J, Tenenbaum J (2015). One shot learning of simple visual concepts. Proc. Cog. Sci. Soc. USA.

[24] Salakhutdinov R, Tenenbaum J, Torralba A (2012). One-shot learning with a hierarchical nonparametric Bayesian model. Proceedings of ICML workshop on unsupervised and transfer learning. USA.

[25] Lin D, Dechter E, Ellis K, Tenenbaum J, Muggleton S (2014). Bias reformulation for one-shot function induction. Proceedings of the twenty-first ECAI. Czech Republic.

[26] Sidman M (1982). A search for symmetry in the conditional discriminations of rhesus monkeys, baboons, and children. Journal of the experimental analysis of behavior.

[27] Markman EM, Wachtel GF (1988). Children’s use of mutual exclusivity to constrain the meanings of words. Cognitive psychology.

[28] Merriman, W. E., Bowman, L. L. & MacWhinney, B. The mutual exclusivity bias in children’s word learning. *Monographs of the society for research in child development* **54** (1989). 2608077

[29] Takahashi T, Nakano M, Shinohara S (2010). Cognitive symmetry: illogical but rational biases. Symmetry: culture and science.

[30] Markman EM (1990). Constraints children place on word meanings. Cognitive science.

[31] Diesendruck G, Markson L (2001). Children’s avoidance of lexical overlap: a pragmatic account. Developmental psychology.

[32] Halberda J (2003). The development of a word-learning strategy. Cognition.

[33] Birch SA, Vauthier SA, Bloom P (2008). Three-and four-year-olds spontaneously use others’ past performance to guide their learning. Cognition.

[34] Jenkins, H. M. & Ward, W. C. Judgment of contingency between responses and outcomes. *Psychological monographs*: *general and applied* **79** (1965). 10.1037/h0093874 14300511

[35] Takahashi T, Oyo K, Shinohara S (2011). A loosely symmetric model of cognition. Advances in artificial life Darwin meets von Neumann. Hungary.

[36] Barrouillet P, Gauffroy C (2015). Probability in reasoning: a developmental test on conditionals. Cognition.

[37] Conway, D. & White, J. Machine learning for hackers. (O’Reilly Media, 2012).

[38] Edgington D (1995). On conditionals. Mind.

[39] Over DE, Evans JSB (2003). The probability of conditionals: The psychological evidence. Mind & Language.

[40] Over DE, Hadjichristidis C, Evans JSB, Handley SJ, Sloman SA (2007). The probability of causal conditionals. Cognitive psychology.

[41] Bayes, M. & Price, M. An essay towards solving a problem in the doctrine of chances. By the late Rev. Mr. Bayes, FRS communicated by Mr. Price, in a letter to John Canton, AMFRS. *Philos*. *Trans*. 370–418 (1763).

[42] Ng, A. Y. & Jordan, M. I. On discriminative vs. generative classifiers: a comparison of logistic regression and naive Bayes. *Advances in neural information processing systems*, 841–848 (2002).

[43] Domingos P, Pazzani M (1997). On the optimality of the simple Bayesian classifier under zero-one loss. Machine learning.

[44] Kwan, K. Y., Lee, T. & Yang, C. Unsupervised n-best based model adaptation using model-level confidence measures. *Seventh International Conference on Spoken Language Processing*, 69–72 (2002).

[45] Salton, G. Automatic text processing: the transformation, analysis, and retrieval of information by computer (Addison-Wesley, 1989).

[46] Katz SM (1996). Distribution of content words and phrases in text and language modelling. Natural language engineering.

[47] Sarkar, A., Garthwaite, P. H. & De Roeck, A. A Bayesian mixture model for term re-occurrence and burstiness. *Proceedings of the Ninth Conference on Computational Natural Language Learning* 48–55 (2005).

